# Shedding new light on an old molecule: quinophthalone displays uncommon N-to-O excited state intramolecular proton transfer (ESIPT) between photobases

**DOI:** 10.1038/s41598-017-04114-9

**Published:** 2017-06-20

**Authors:** Gi Rim Han, Doyk Hwang, Seunghoon Lee, Jong Woo Lee, Eunhak Lim, Jiyoung Heo, Seong Keun Kim

**Affiliations:** 1 Seoul National University, Department of Chemistry, Seoul, 08826 Republic of Korea; 2 Seoul National University, Department of Biophysics and Chemical Biology, Seoul, 08826 Republic of Korea; 3 Sangmyung University, Department of Biomedical Technology, Chungnam, 31066 Republic of Korea

## Abstract

Excited state dynamics of common yellow dye quinophthalone (QPH) was probed by femtosecond transient absorption spectroscopy. Multi-exponential decay of the excited state and significant change of rate constants upon deuterium substitution indicate that uncommon nitrogen-to-oxygen excited state intramolecular proton transfer (ESIPT) occurs. By performing density functional theory (DFT) and time-dependent density functional theory (TDDFT) calculations, we found that adiabatic surface crossing between the S_1_ and S_2_ states takes place in the photoreaction. Unlike most cases of ESIPT, QPH does not exhibit tautomer emission, possibly due to internal conversion or back-proton transfer. The ESIPT of QPH presents a highly interesting case also because the moieties participating in ESIPT, quinoline and aromatic carbonyl, are both traditionally considered as photobases.

## Introduction

ESIPT, where migration of a proton takes place within a molecule after photoexcitation, is a fundamental photophysical process that garnered decades of interest in varying fields of chemistry. Widespread attention to the phenomenon stems from the fact that molecules that undergo ESIPT may exhibit emission with a large Stokes’ shift, which minimizes self-absorption and opens up possibilities of diverse applications^1^. Some examples utilizing this property include molecular sensors^2^, optical memory^3^, and white light sources^4^.

Most research on ESIPT utilizes tautomer fluorescence for probing dynamics^5–7^ and therefore lack of such emission, as is the case with our subject molecule QPH, poses challenge to investigation of ESIPT. Another problem is that it is difficult to recognize ESIPT at first sight if the tautomer is non-emissive. Possibility of fully non-radiative ESIPT has only recently been recognized by Yin *et al*.^8^ and we suspect that this is the reason why ESIPT dynamics of QPH went unnoticed until now.

**Figure 1:**
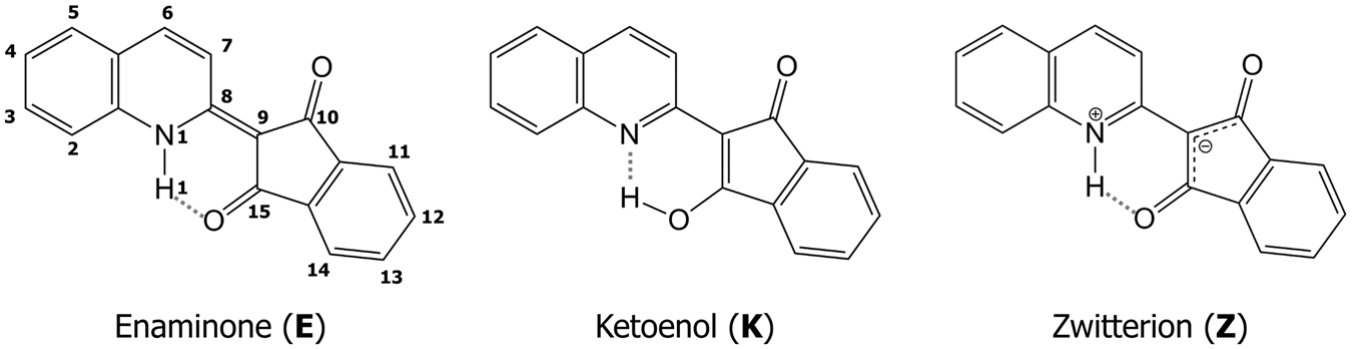
Possible tautomers of QPH.

QPH, or more commonly known as quinoline yellow, is a commercial dye with distinctive greenish yellow color. It has three possible tautomers (Fig. 1), of which the enaminone (**E**) form is confirmed to be the most stable one by NMR^9, 10^ and calculation^11^. Unlike the ketoenol (**K**) and zwitterion (**Z**) forms, the bond connecting phthalone and quinoline rings is a double bond, making the molecule nearly coplanar^12^. An intramolecular hydrogen bond (IMHB) exists between hydrogen attached to the nitrogen of quinoline and the carbonyl group of the phthalone moiety. Previous researchers acknowledged possible contribution of the **Z** form in the ground state, whereas the **K** form does not appear in NMR and is believed to be too energetically unstable (~30 kJ/mol) to exist in the ground state.

QPH has a long history of usage since it was discovered in 1882^13^. Because of its many desirable traits such as high solubility, significant resistance against photodegradation, and easy synthesis, QPH and its derivatives are used as coloring agents for various materials including polyester fibers, wools, paper, wax, paraffin, paints, and silk^13–16^. Certification of QPH and its sulfonated derivative by the European Union and FDA allowed more diverse applications.

Until now the majority of research on QPH has been limited to identifying potential cellular toxicity and developing assays for analysis. Especially the matter of its biocompatibility has long been a subject of debate^17–19^. However, to the best of our knowledge, there exists only one photodynamics study of the molecule in pH 12 water conducted in the nanosecond scale^15^. Results in organic solvent and neutral pH water were not reported because the authors found no interesting signal. This most likely indicates that de-excitation dynamics of QPH is completed within sub-nanosecond scale.

We hypothesized that QPH’s notable stability against light may originate from its ultrafast relaxation to the ground state after photoexcitation and attempted to carry out femtosecond pump-probe spectroscopy in order to unravel the long-neglected picture of QPH photochemistry. In doing so we have unexpectedly discovered signs of intramolecular proton transfer occurring in the excited state.

In the following sections, we report evidence of QPH displaying an unprecedented case of ESIPT between two groups known to be photobases (quinoline and aromatic carbonyl) and provide theoretical basis for its occurrence in QPH.

## Results

### Absorption and emission spectra

**Figure 2:**
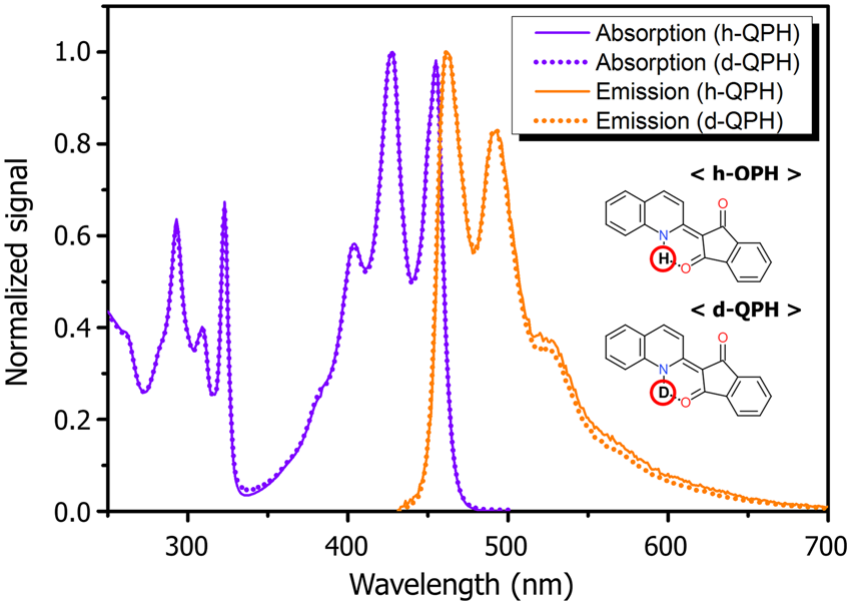
Absorption/emission spectrum of QPH (h-QPH) and deuterated QPH (d-QPH) in cyclohexane.

QPH is known for negative solvatochromism, i.e., absorption peaks shifting to shorter wavelengths in polar solvents^14, 20^. It is a common behavior for many polar organic molecules that have environment-sensitive energy levels. Strong absorption below 460 nm gives QPH its characteristic yellow hue. Although emission in polar solvent is virtually nonexistent, we could observe weak photoluminescence in highly nonpolar solvents such as cyclohexane (Fig. 2). Even then, the emission signal is very low, reaffirming that significant non-radiative process is involved in relaxation dynamics of the molecule. A small Stokes’ shift and near-mirror image symmetry of the absorption vs. emission peaks indicate that the emission is most likely fluorescence from the lowest singlet excited state^21^.

### Transient absorption spectroscopy

**Figure 3:**
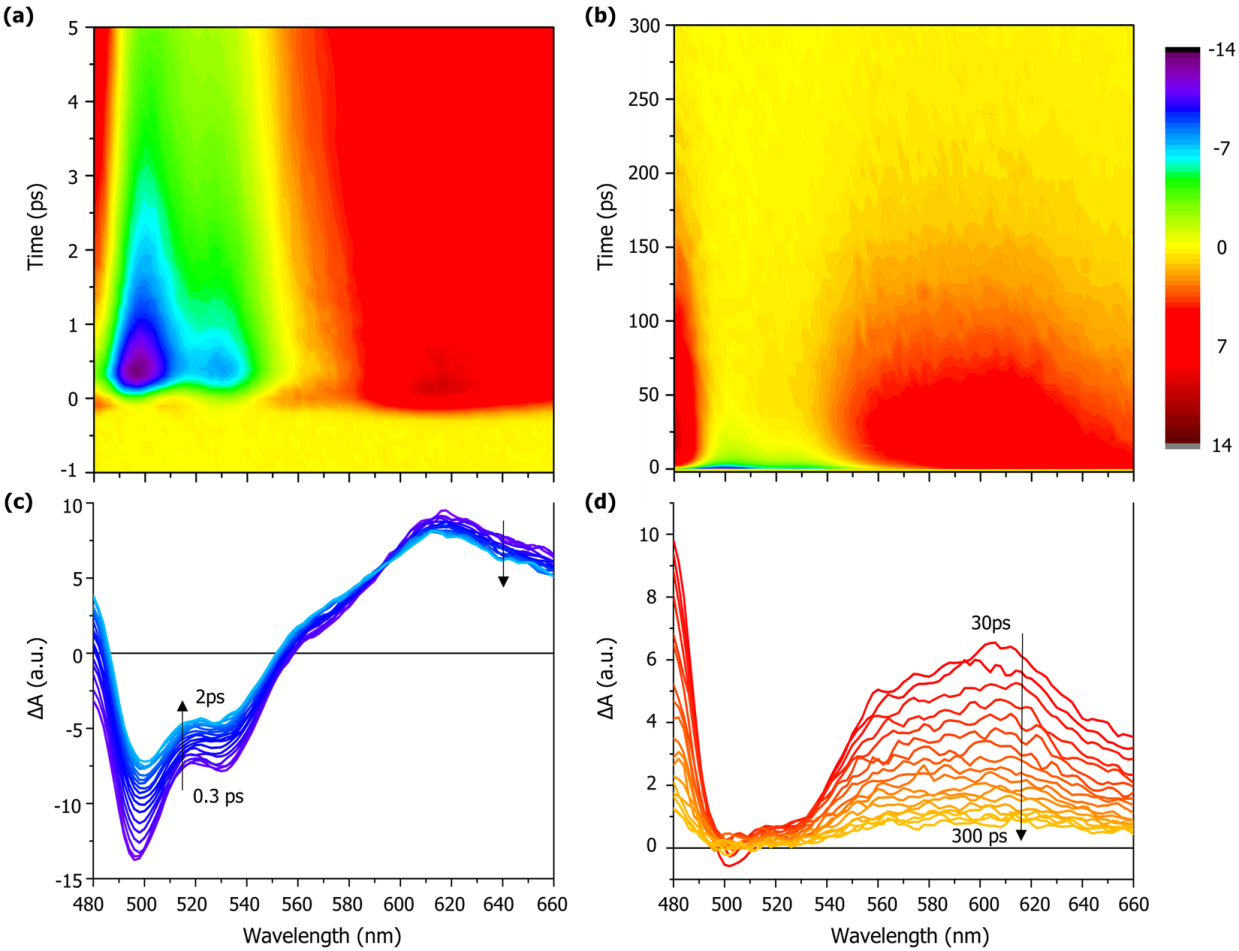
Transient absorption spectrum of QPH in cyclohexane (100 μM). (**a**) and (**b**) show 3D plots in different time scales, whose spectral evolution is shown in (**c**) from 0.3 to 2 ps and (**d**) from 30 to 300 ps.

To probe the ultrafast relaxation process of QPH, we conducted femtosecond transient absorption measurement. Figure 3 shows transient absorption spectrum of QPH in cyclohexane excited with 400 nm pump and probed with supercontinuum light in the range of 480 to 660 nm. It is apparent from the picture that the decay kinetics cannot be represented by a single exponential curve, indicating the possibility of relaxation involving more than one excited state. We also take note of the fact that the spectra in the shorter timescales (Fig. 3c) show a “dynamic isosbestic point” near 590 nm, where absorbance remains the same regardless of the temporal evolution. It is generally regarded as a sign that a transient species is evolving into another over the course of time, rather than there being two unrelated excited species^22^. After rapid initial decay, the time profile shows a complex trace for tens of ps, after which the decay becomes nearly single exponential (Fig. S1). Almost no signal is found after 300 ps, indicating that most species has returned to the ground state.

Lifetimes that best explain temporal behavior at four different wavelengths of 500, 530, 600, and 620 nm are acquired by global nonlinear fit (Fig. S1) and presented in Table 1. As the current decay profiles are not compatible with simple exponential fit, we truncated their middle part and divided them into a single exponential head (<2 ps) and a biexponential tail (>30 ps) for separate analysis. The initially populated state shows a strong negative signal below 580 nm, which may be interpreted as either disappearance of the ground state species or increased photon output due to stimulated emission^23^. Because the absorption spectrum of ground state QPH ends around 450 nm and the emission spectrum has peaks at similar wavelengths, it is most likely to be stimulated emission signal. Therefore the initially decaying species is presumably the lowest singlet excited state, which is an observable emissive state. From the rapid decay, we know that the emissive state lasts very short, which may explain the low quantum yield of the molecule. From the aforementioned isosbestic point, we assume that it evolves into a different excited state. The excited state absorption signal peaked at 610 nm lasts longer (until 300 ps), suggesting that at least two or more states are involved in the process.

**Table 1:** Lifetimes (in ps) of h-QPH and d-QPH and their ratios.

	h-QPH	d-QPH	**τ**_D_/**τ**_H_ (=k_H_/k_D_)
**τ**_1_	3.268 (±0.29)	4.53 (±0.41)	1.39
**τ**_2_	15.26 (±1.57)	16.8 (±2.03)	1.10
**τ**_3_	83.91 (±1.45)	112.3 (±2.53)	1.34

**τ**_**1**_ was determined from single exponential fitting of the sub-2 ps profile, while **τ**_**2**_ and **τ**_**3**_ were determined from biexponential fitting of over-30 ps profile. (*Standard error in parenthesis).

### Deuterium isotope effect

**Figure 4:**
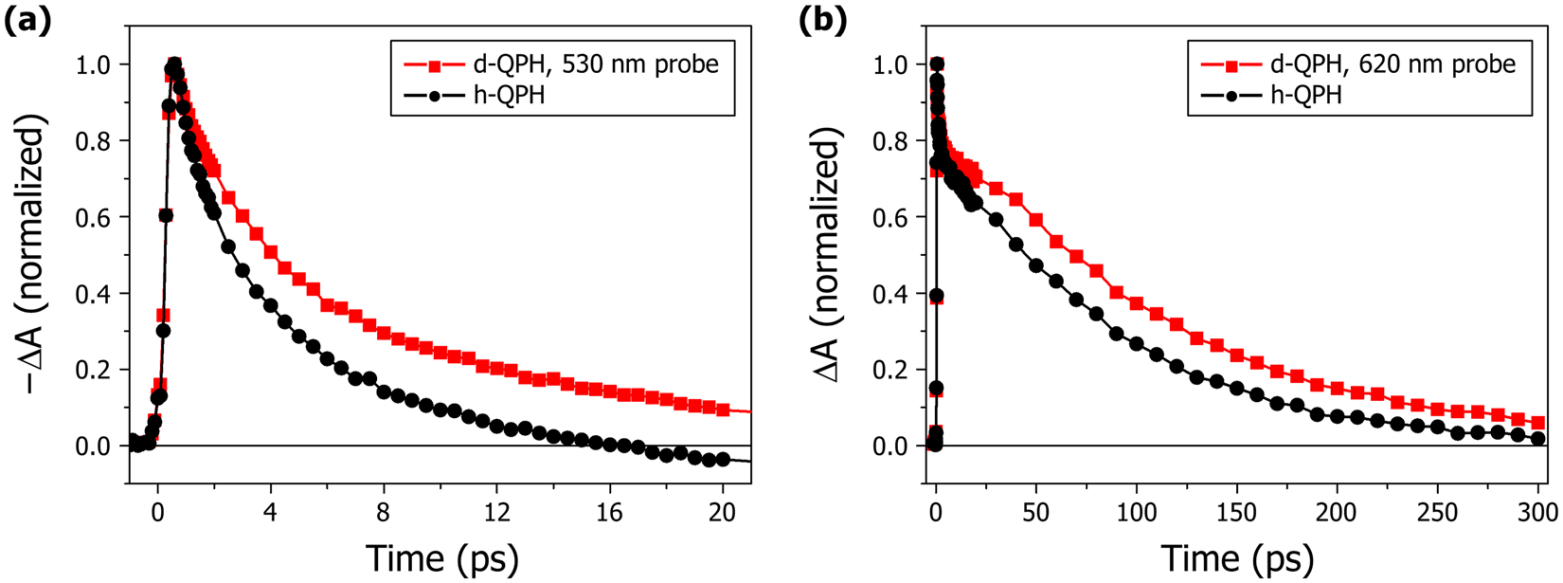
Comparison of time profiles between deuterium substituted QPH and unsubstituted QPH at the probe wavelength of (**a**) 530 nm and (**b**) 620 nm.

To identify the evolutionary course of the excited state, we carried out comparative experiments using deuterium substituted QPH and unsubstituted QPH. As the hydrogen participating in IMHB (H_1_) is known to be acidic^14^, we substituted it with deuterium (d-QPH) by following the protocols in the Methods section and acquired time profile at several wavelengths. The same procedure was carried out using water (h-QPH) and compared against QPH solution in cyclohexane to ensure that it was not the effect of hydrate formation. Overall spectral features of d-QPH remained similar to those of h-QPH, including stimulated emission and the isosbestic point, with their only difference being the decay rate. Comparison of the time profiles between d-QPH and h-QPH readily shows significantly slower kinetics of the former (Fig. 4). Lifetimes in Table 1 indicate that a primary kinetic isotope effect may be present in both **τ**_**1**_ and **τ**_**3**_.

Since isotopic mass change does not affect the force field of the molecule, we expect that only the nuclear motions involving the substituted atom will change^23^. In fact, deuterium substitution has been extensively used to verify ESIPT^6^. As the mass of H_1_ hydrogen comprises a very small fraction of the mass of QPH, it is unlikely that single deuterium substitution can significantly affect any motion other than the N-H vibration. Since the vibrational frequency of an oscillator is inversely proportional to the square root of the reduced mass of the oscillator if the force constant remains unchanged, we expect the ratio **τ**_**D**_/**τ**_**H**_ ( = **k**_**H**_/**k**_**D**_) of Table 1 should be nearly equal to the square root of the ratio of the reduced masses between N-D and N-H, or {[(14 × 2)/(14 + 2)]/[(14 × 1)/(14 + 1)]}^1/2^ = (15/8)^1/2^ = 1.37, which is actually in very good accord with our measured values for **τ**_**1**_ and **τ**_**3**_, indicating that it is indeed the motion of hydrogen such as occurring in ESIPT that governs the relaxation of the excited state.

### Quantum calculation using DFT/TDDFT

**Figure 5:**
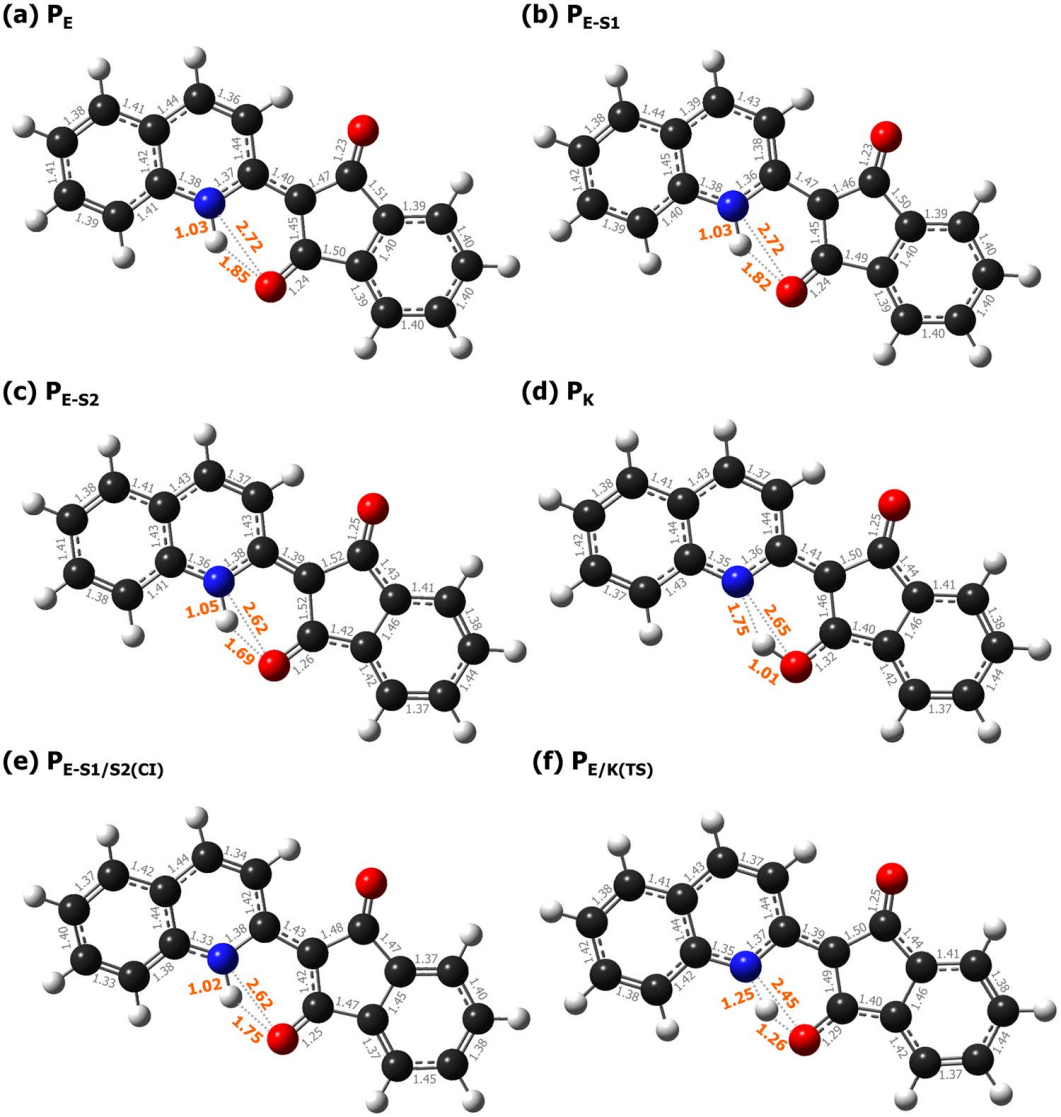
Point geometry of (**a**) ground state of **E** form, (**b**–**d**) local minima at the first singlet excited state, (**e**) conical intersection between S_0_/S_1_ of the **E** form, (**f**) transition state between the **E** and **K** tautomer (units in angstrom (Å)).

**Figure 6:**
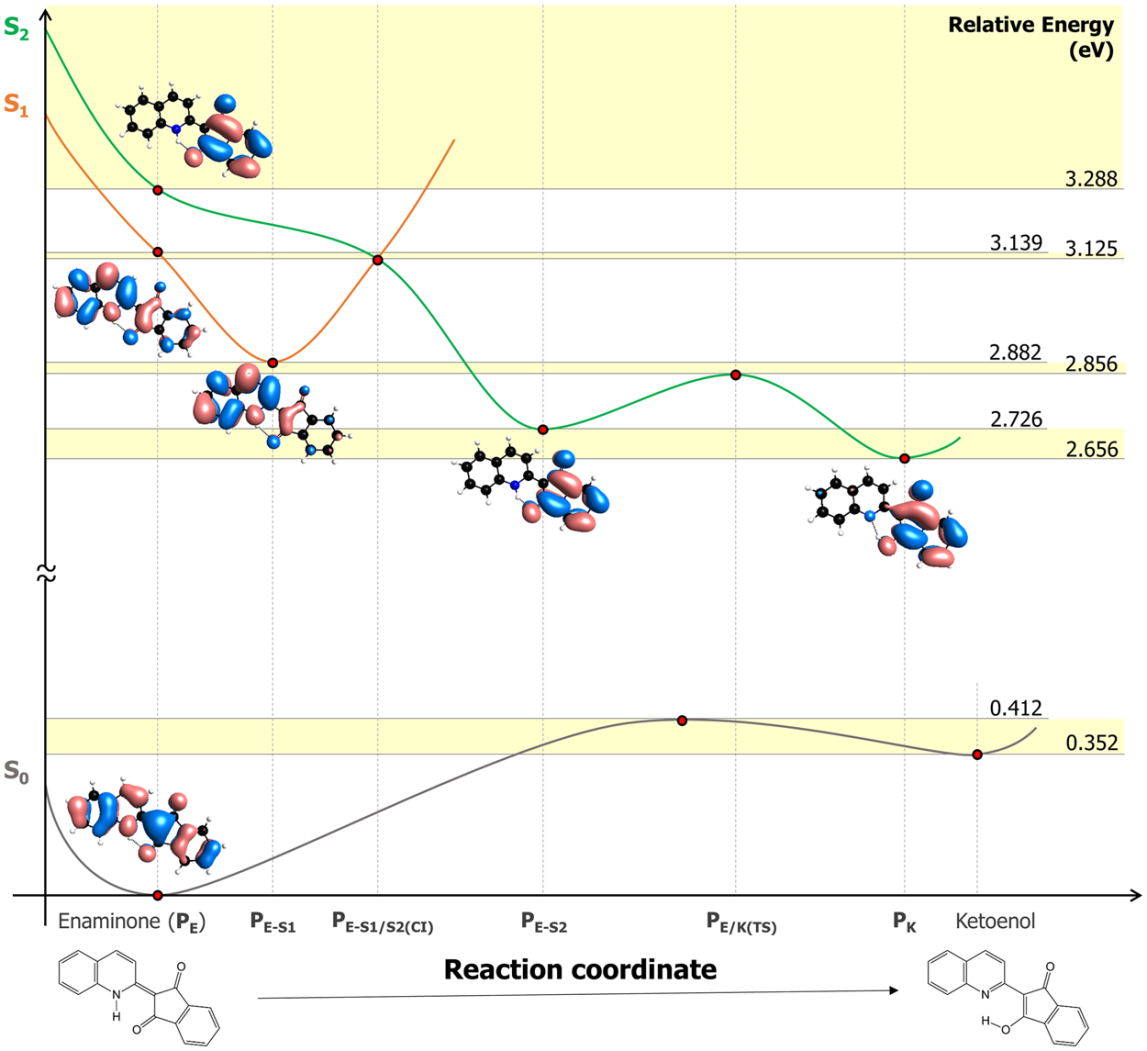
Schematic diagram of QPH species with relevant geometries and molecular orbitals in the S_0_, S_1_ and S_2_ states.

Since ESIPT in systems like QPH is unexpected and rare as previously mentioned, we investigated the dynamics of ESIPT by DFT/TDDFT calculations. In order to enlist different QPH species involved in the reaction, we first define the optimized geometry of the singlet ground state (S_0_) of the **E** tautomer as **P**_**E**_ (Fig. 5(a)). This is the geometry where the HOMO, LUMO and LUMO+1 shown in the leftmost side of Fig. 6 constitutes a major MO for the S_0_, S_1_ and S_2_ states, respectively. Optimizing the first excited state at the Franck-Condon point **P**_**E**_ yields **P**_**E-S1**_ (Figs 5(b) and 6), the state property of which differs markedly from that of the experimentally expected **K** tautomer, **P**_**K**_ (Fig. 5(d)). This led us to suspect a missing link and search for other, more relevant local minima on the S_1_ potential energy surface along the reaction coordinate. We eventually found another local minimum located between **P**_**E-S1**_ and **P**_**K**_, which is denoted **P**_**E-S2**_ (Fig. 5(c)) as it is associated with the S_2_ state (see below).

Each geometry possesses distinctive structural and electronic properties as described below.

- The LUMO at **P**_**E-S1**_ is very similar to that at **P**_**E**_, indicating that the former is a relaxed conformation of S_1_ after vertical excitation, hence the designation **P**_**E-S1**_. The bond lengths of N_1_−H_1_ and O_15_···H_1_ are nearly identical between **P**_**E-S1**_ and **P**_**E**_, indicating little change in the interaction between atoms directly involved in IMHB.
- The LUMO at **P**_**E-S2**_ is similar to the LUMO+1 (S_2_ state) at **P**_**E**_ (Fig. 6), hence the designation **P**_**E-S2**_. The bond length of N_1_−H_1_ increases only very slightly, but the O_15_···H_1_ and N_1_···O_15_ distances become noticeably shorter.
- **P**_**K**_ is the proton-transferred geometry (**K** form). Striking similarities between its LUMO and the LUMO+1 at **P**_**E**_ and the LUMO at **P**_**E-S2**_ are apparent in Fig. 6.

**P**_**E-S2**_ is the key intermediate geometry between **P**_**E**_ and **P**_**K**_, as its tautomeric structure is similar to the geometry at **P**_**E**_ but its LUMO is similar to that at **P**_**K**_. Also, the LUMO at **P**_**E-S2**_ lies lower in energy than LUMO at **P**_**E-S1**_ but higher than that at **P**_**K**_, making it a more likely candidate for a geometry immediately preceding proton transfer.

Assigning the S_1_ state at **P**_**E-S2**_ as the reaction intermediate leads us to two assumptions about the potential energy surface. First of all, because the electron density of the LUMO shifts from the quinoline ring at **P**_**E**_ and **P**_**E-S1**_ to the phthalone ring at **P**_**E-S2**_ and **P**_**K**_, we may suspect the presence of an adiabatic surface crossing between the S_1_ and S_2_ excited states, which is also consistent with the similarity between the LUMO+1 at **P**_**E**_ and the LUMO at **P**_**E-S2**_ (see also Figure S2 to observe apparent ‘flip’ in molecular orbitals). From the state property of each species, we deduce that the lowest singlet excited states of **P**_**E**_ and **P**_**E-S1**_ are on a same adiabatic surface while those of **P**_**E-S2**_ and **P**_**K**_ are on another adiabatic surface. In order to see the possibility of the nonadiabatic transition, we searched for a conical intersection of the S_1_ and S_2_ surfaces and found one at **P**_**E-S1/S2(CI)**_ (Fig. 5(e)). Although the calculated value did not take the solvent effect into account, the transition is still shown to be energetically feasible when considering the excess excitation energy. Also the geometry at **P**_**E-S1/S2(CI)**_ is quite similar to those at **P**_**E-S1**_ and **P**_**E-S2**_ (Fig. 5), which implies no additional barriers to the conical intersection.

Another assumption is that the transition state (TS) from **E** to **K** is expected via the **P**_**E-S2**_ geometry. By placing H_1_ midway between N_1_ and O_15_, we found what appears to be a transition state (**P**_**E/K(TS)**_ in Fig. 5(e)) that has a low energy barrier of ~0.13 eV (Fig. 6). Intrinsic reaction coordinate (IRC) path has been determined starting from **P**_**E/K(TS)**_ by the steepest descent method, which led to minima that correspond to **P**_**E-S2**_ and **P**_**K**_ (Fig. S3). Therefore **P**_**E-S2**_ is deemed the doorway geometry for the proton transfer.

From the results above we construct a rudimentary potential energy surface diagram shown in Fig. 6. QPH photoexcited at the Franck-Condon point (**P**_**E**_) will vibrate about the local minimum **P**_**E-S1**_ on the S_1_ adiabatic surface, during which a nonadiabatic transition to the S_2_ state occurs at **P**_**E-S1/S2(CI)**_, which leads to the ESIPT process toward the point **P**_**K**_ via **P**_**E-S2**_ and **P**_**E/K(TS)**._ We also note that we could not find a conical intersection between S_1_ and S_0_ of the **K** tautomer.

Natural bond orbital (NBO) charge analysis reveals that N_1_ becomes electrostatically less negative (−0.520 to −0.488) and O_15_ more negative (−0.617 to −0.656) in the S_1_ state at **P**_**E-S2**_ relative to those of the S_1_ state at **P**_**E**_ (Fig. S4). Such a change in electrostatic charge of atoms involved in IMHB should be a preceding sign of ESIPT.

## Discussions

Molecules known for ESIPT typically display either a large Stokes’-shifted emission or dual emission^7^. Despite the fact that both characteristics are absent from the spectrum in Fig. 1, our experiment on deuterium substitution of H_1_ proton verified the occurrence of ESIPT in QPH. Therefore we may conclude that after ESIPT, the **K** form of QPH undergoes a significant nonradiative decay. There are some notable examples for ESIPT that do not display tautomer emission. One is hypericin, for which it was speculated that either energy levels remain unchanged upon tautomerization or that tautomers are mixed in the ground state^24^. Another is 1-hydroxypyrene-2-carbaldehyde, for which intersystem crossing to a triplet state happens^8^. However, from aforementioned calculations and NMR results, we may rule out the possibility of the ground state tautomerization. For the latter possibility, we found the time profile of QPH insensitive to nitrogen purging (Fig. S5) and thus intersystem crossing to a triplet state seems unlikely.

From our experimental results for deuterium substitution and the lack of conical intersection between S_1_ and S_0_ of the **K** tautomer, we suspect that the vibrational mode related to IMHB is involved when the **K** tautomer returns to the ground state. It has been discussed in depth that the presence of hydrogen bond is associated with enhanced internal conversion back to the ground state and that the isotope effect may appear if the associated hydrogen is deuterated^25–28^. For ESIPT, some cases are known where enhanced internal conversion lowers photoluminescence quantum yield after tautomerization^6, 29^. Another possibility is that ultrafast relaxation occurs via back proton transfer process^5^. Currently we cannot differentiate between the two possibilities and will leave it for future studies.

It is also interesting to note that the biexponential global fit after 30 ps brings about a **τ**_2_ component of ~15 ps that is seemingly unaffected by deuteration. This observation, along with the complex nature of kinetic traces before 30 ps, suggests that there might be another relaxation pathway other than ESIPT.

Although IMHB is an important factor in facilitating excited state intramolecular hydrogen transfer, not every molecule with IMHB undergoes ESIPT. It has been well-established that ESIPT occurs readily in conjugated ketoenol system (O···HO) or, similarly, N···HN system and frequently reported, but ESIPT between heteroatoms are less known^30^. Especially, ESIPT from nitrogen to oxygen is a rare case, but not completely unheard of. Some molecules known for displaying N-to-O ESIPT include *o*-acetylaminoacetophenone^25^ and 1-(acylamino)anthraquinone with appropriate functional groups^31^. More cases are known if dimerized molecules are considered^32^. However, what makes our case unique is that the proton donor of QPH is quinoline, which is better known for its photobasicity, meaning that it is more likely to accept a proton in the excited state^33^.

The driving force for ESIPT has been traditionally explained by the change in electron density at atoms involved in ESIPT. Excited state tautomerization is usually accompanied by electron redistribution after vertical excitation^34^, and the resulting change in the relative acidity and basicity is explained by the change in electron density at ‘heavy atoms’ (nitrogen or oxygen)^35^. Calculations of electrostatic charge at atoms related to photoacidity have been presented as supporting evidence in several studies of established systems^33, 36^. Results of our NBO calculation may be interpreted in similar way. In the transient **P**_**E-S2**_ state, the increased electron density renders carbonyl oxygen O_15_ photobasic, and the decreased electron density makes N_1_ photoacidic. Because acidity and basicity are only relative terms, we suggest that if the relative strengths differ, ESIPT between photobases may occur.

Usually the magnitude of deuterium isotope effect is assessed by parameter k_H_/k_D_, whose value may lie anywhere between 1 and 50, depending on the shape of potential energy surface and the size of energy barrier^34, 37^. Furthermore, the kinetic isotope effect may not appear at all when the system reaches the adiabatic limit. Generally, such lack of isotope effect is regarded as a sign of unsubstantial potential energy barrier, and happens in cases when the distance between heavy atoms involved in ESIPT is sufficiently short^5, 29^. Since our system displays a clear isotope effect, the possibility of a barrierless potential energy surface may be excluded. Also the k_H_/k_D_ value of 1.39 and 1.35 in our system is nearly identical to the square root of the reduced mass ratio between hydrogen and deuterium, suggesting the crucial role of H_1_ vibration on the overall kinetics.

To summarize, we conducted ultrafast transient absorption spectroscopy of QPH and observed signs of ESIPT. This study is the first report to observe the ESIPT of QPH that involves a very rare case of proton transfer from nitrogen to oxygen. Also, this study reports the first case of ESIPT between two traditionally known photobasic moieties, quinoline and aromatic carbonyl^35^. Calculated results show that an S_1_-S_2_ adiabatic surface crossing occurs before undergoing ESIPT.

## Methods

Quinophthalone (Quinoline Yellow 2SF, ≥97%, #01354) was purchased from Sigma-Aldrich and used without further purification. All solutions were prepared using ACS grade cyclohexane (Sigma-Aldrich, #179191) with 100 μM concentration.

The d-QPH sample was prepared by sonicating the 1:2 volume mixture of QPH solution and D_2_O (Sigma-Aldrich, #151882) in the same vial. Then we waited for separation and took the upper layer of cyclohexane. We tested several experimental conditions and found that 15 minutes of sonication and 90 minutes of waiting for separation were enough to reproduce the results. The same procedure was adopted with HPLC grade water (JT Baker, #4218–03) to ensure that the change originates from the isotope effect. ^1^H NMR spectrum of the solutions (Fig. S6) show that the majority of H_1_ proton is deuterated by the protocol.

Steady-state UV/Vis absorption spectra and photoluminescence spectra were recorded using Lambda 25 (Perkin-Elmer) and QM-3/2004SE (PTI), respectively.

Femtosecond transient absorption spectroscopy was conducted using conventional pump-probe setup. The optical source was a regeneratively-amplified Ti:Sapphire laser (Spectra-Physics) with an average power of 0.65 mJ/pulse, pulse width of 130 fs FWHM, and repetition rate of 1 kHz at 800 nm output wavelength. Pump pulses were generated using a second harmonic generator (TP1A, Spectra-Physics), filtered down by neutral density filter and used at 1.5 mW. A continuum light, which was used as the probe pulse, was generated by focusing the 800 nm light using a UV-fused silica plano convex lens (CVI Melles Griot, f = 100 mm) onto a sapphire plate (WG31050, Thorlabs). Diameters of the pump and probe beams at the sample position were 390 μm and 295 μm, respectively. The IRF (~250 fs FWHM) was estimated by the cross-correlation between the pump with the probe. Solvent background subtraction and GVD-correction were done according to the known methods^38^. The time delay between the pump and the probe pulses was scanned using Daedal 404300XRMP delay stage (Parker). The pump pulses were modulated using an optical chopper (MC1000, Thorlabs) synchronized to the laser. The probe pulses of the signal were detected by photodiodes (2031, New Focus) after wavelength selection using a monochromator (250 IS/SM, Chromex, 600 grooves/mm grating blazed at 750 nm). Transient absorption signals were obtained using a lock-in amplifier (SR830, Stanford Research Systems). Temperature was controlled at 23 °C.

Calculations were executed using the DFT and TDDFT methods at B3LYP/6-31 G(d) level of theory. Structure optimization was performed using quadratic approximation with gradient convergence tolerance of 0.0001 Hartree/Bohr (default value) using General Atomic and Molecular Electronic Structure System (GAMESS)^39, 40^ and Gaussian 09^41^. Transition state and conical intersection were acquired using the default method in GAMESS. It must be noted that the branching plane update method for CI search requires TAMMD approximation and therefore was applied only on this value. It is known that it gives a better intermediate geometry during photophysical processes^42^. NBO charge analysis was performed with the NBO version 3.1 included in Gaussian 09^43^. Molecular structures and orbitals were visualized with GaussView 5.0.8^44^ and wxMacMolPlt^45^.

## Electronic supplementary material

Supplementary Materials
