# Supplementary material for: Shedding new light on an old molecule: quinophthalone displays uncommon N-to-O excited state intramolecular proton transfer (ESIPT) between photobases
Source: Sci Rep. 2017 Jun 20;7:3863. doi: 10.1038/s41598-017-04114-9 (PMC5478638; doi:10.1038/s41598-017-04114-9)
Supplement: Supplementary file 1 — Supplementary Materials [file 41598_2017_4114_MOESM1_ESM.pdf]

Supplementary Materials

Shedding new light on an old molecule: quinophthalone displays uncommon N-to-O excited state intramolecular proton transfer (ESIPT) between photobases

Gi Rim Han[1, +], Doyk Hwang[2, +], Seunghoon Lee[1], Jong Woo Lee[2], Eunhak Lim[1], Jiyoung Heo[3], and Seong Keun Kim[1, 2, *]

[1]Seoul National University, Department of Chemistry, Seoul 08826, Republic of Korea
[2]Seoul National University, Department of Biophysics and Chemical Biology, Seoul 08826, Republic of Korea
[3]Sangmyung University, Department of Biomedical Technology, Chungnam 31066, Republic of Korea
[*]Corresponding: seongkim@snu.ac.kr
[+]these authors contributed equally to this work

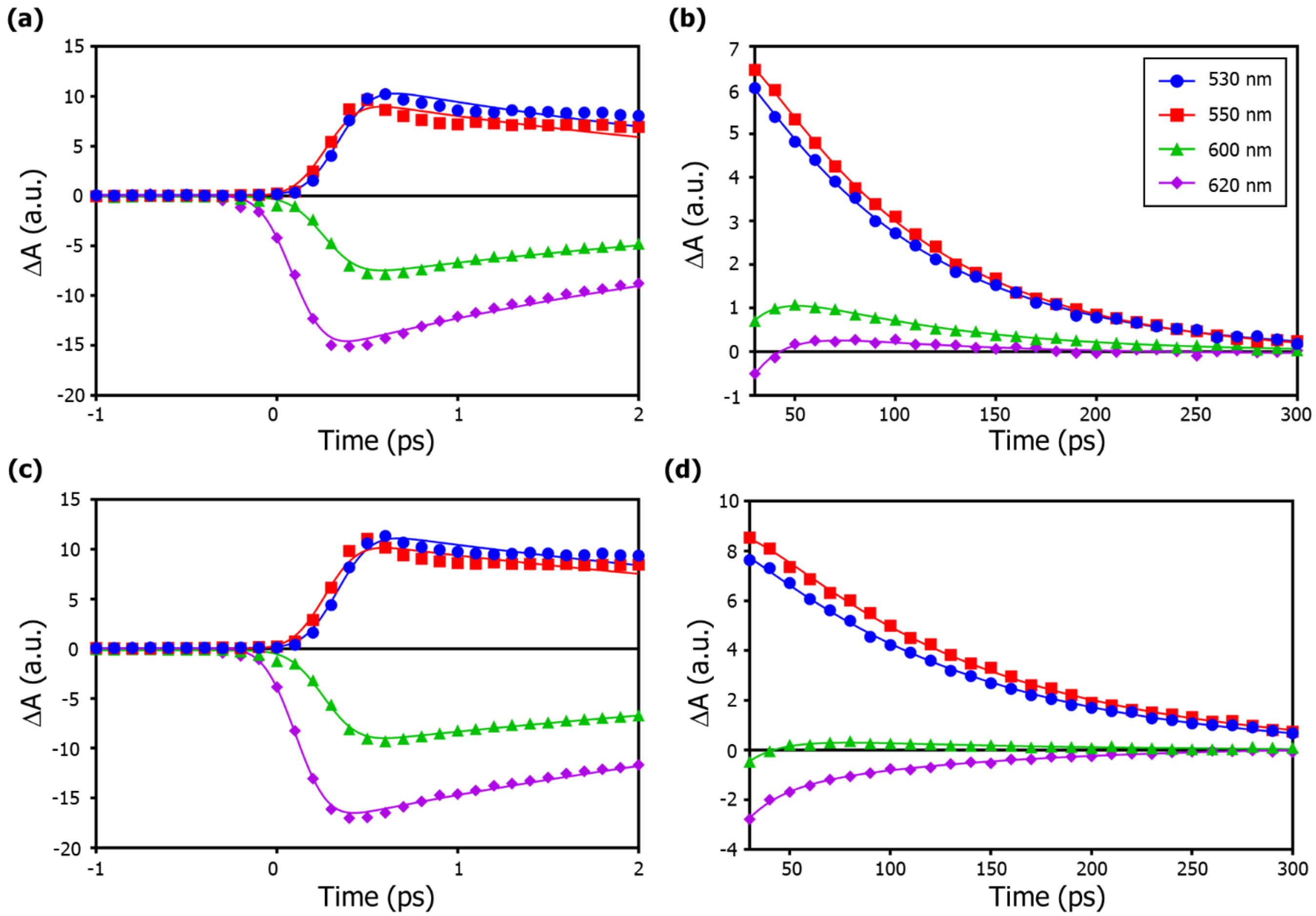

Figure S1. Raw data and global fit of time profiles at four different probe wavelengths for (a, b) h-QPH in cyclohexane (100 μM) and (c, d) d-QPH in cyclohexane (100 μM). Note that these profiles are not GVD-corrected unlike other data in the paper. Different time zeros are included explicitly in the fitting functions.

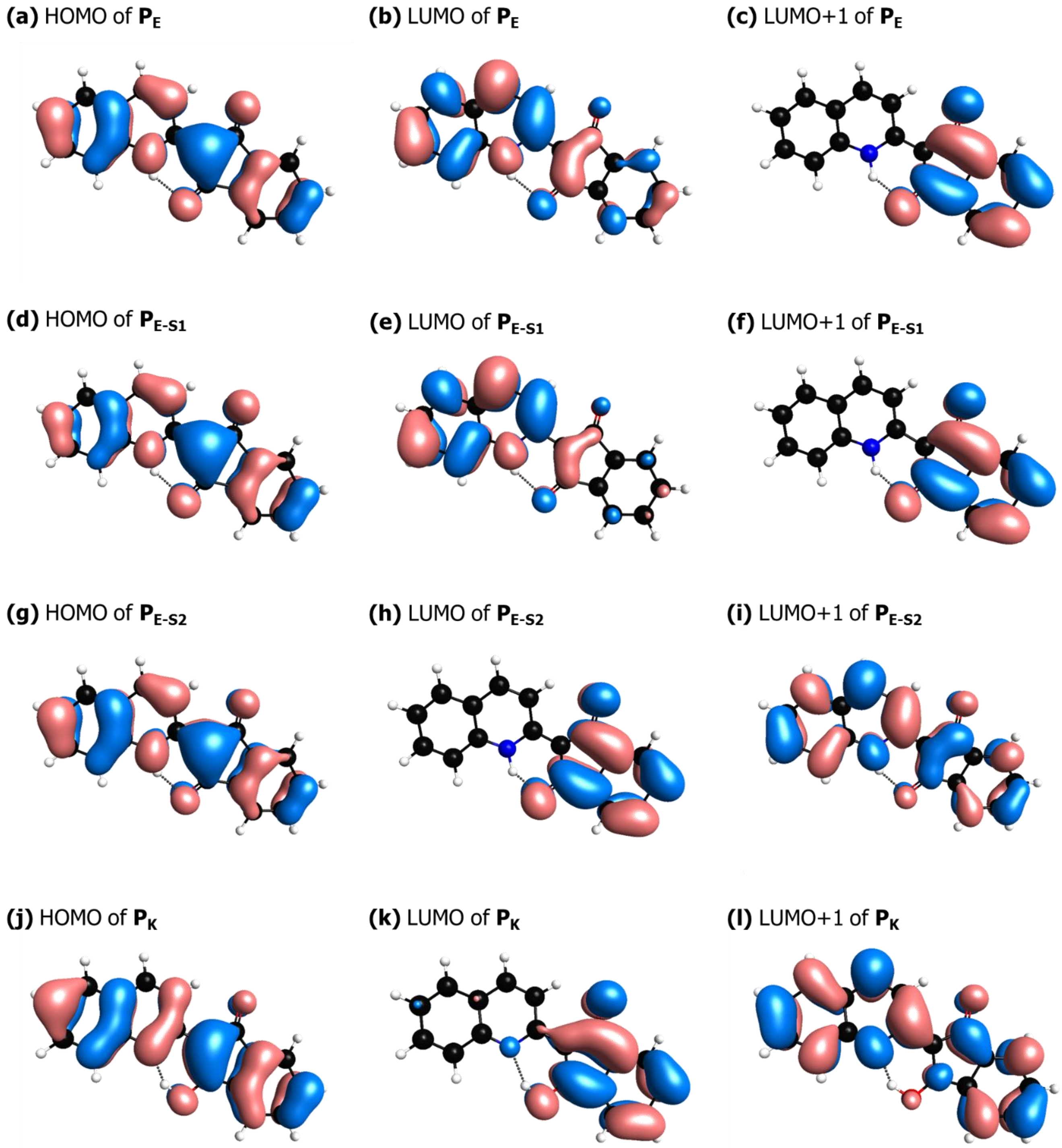

Figure S2. HOMO, LUMO and LUMO+1 at (a - c) $\mathbf{P}_{\mathbf{E}}$, (d - f) $\mathbf{P}_{\mathbf{E-S1}}$, (g - h) $\mathbf{P}_{\mathbf{E-S2}}$ and (j - l) $\mathbf{P}_{\mathbf{K}}$.

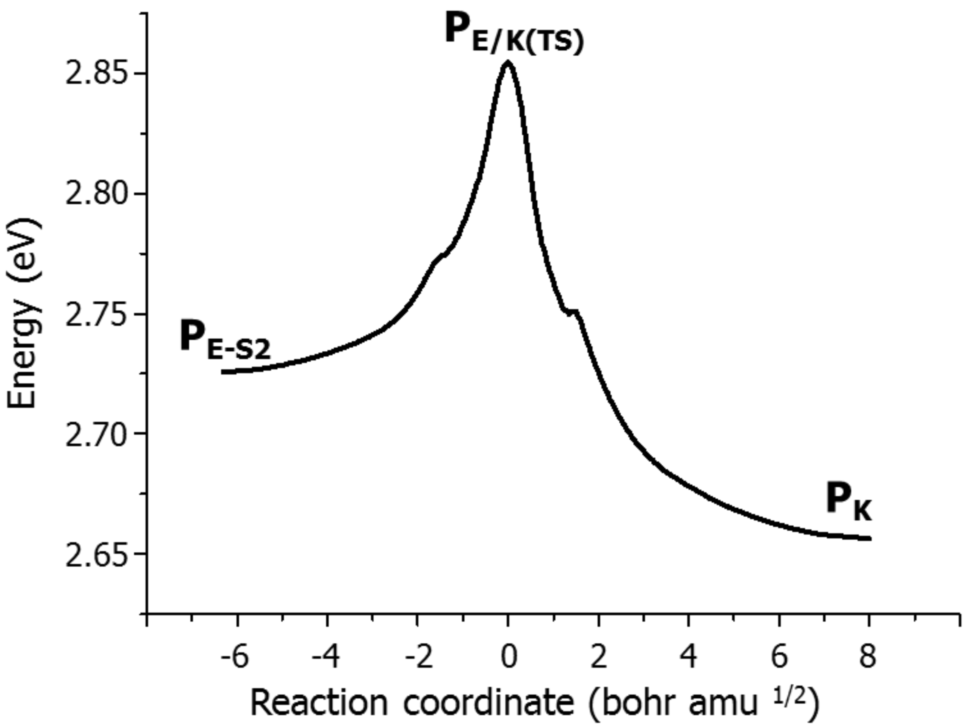

Figure S3. Intrinsic reaction coordinate gained from $\mathbf{P_{E/K(TS)}}$ geometry using the steepest descent method. The energy level is assigned in respect to the ground state energy level of $\mathbf{P_E}$.

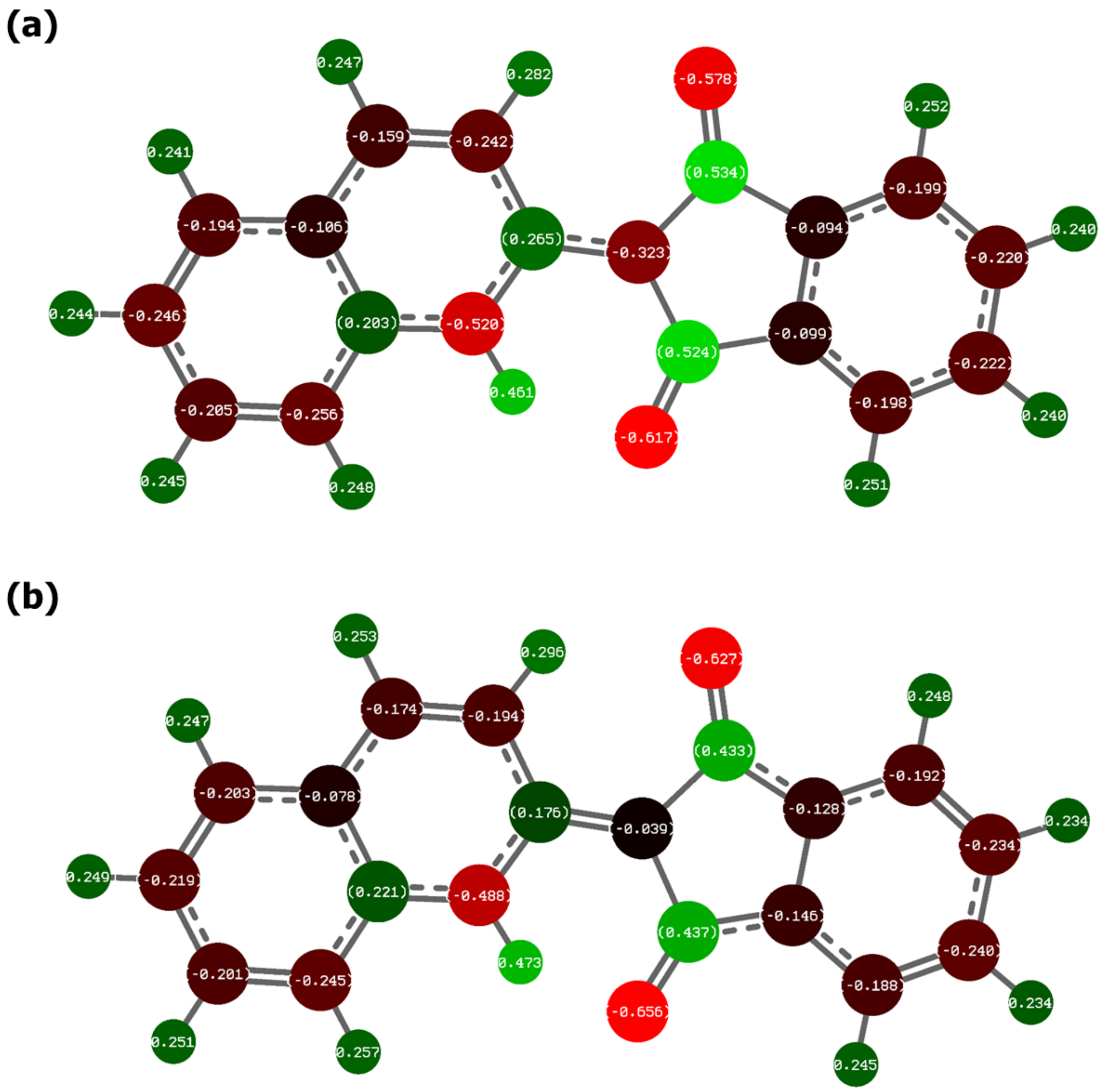

Figure S4. NBO charges in (a) the ground state at $\mathbf{P_E}$ and (b) the first excited state at $\mathbf{P_{E\text{-}S2}}$.

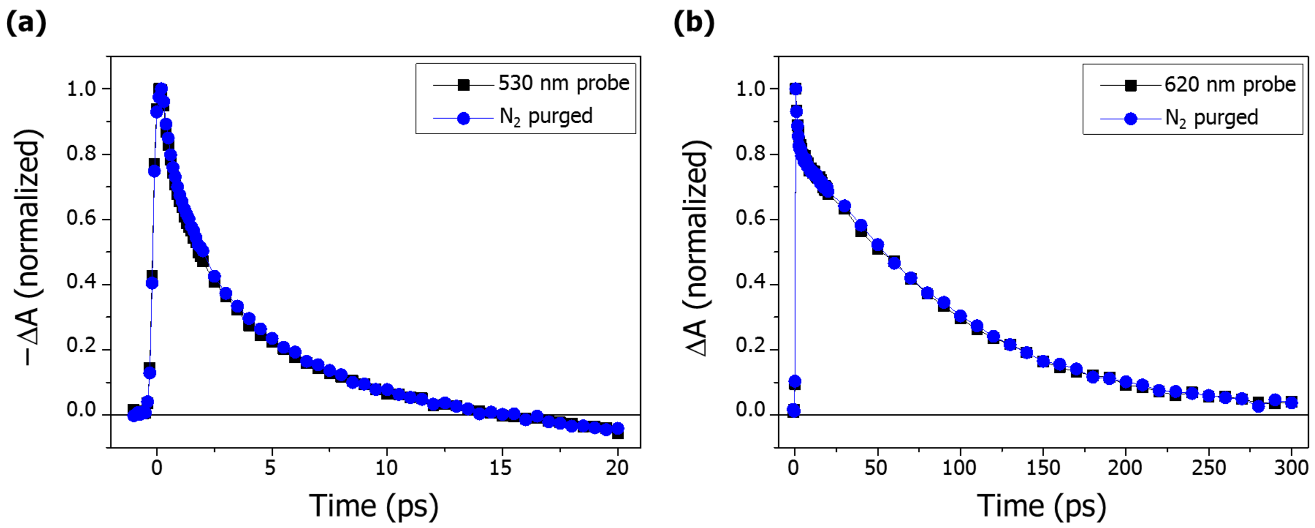

Figure S5. Transient time profile before and after nitrogen purging at probe wavelengths of (a) 530 nm and (b) 620 nm.

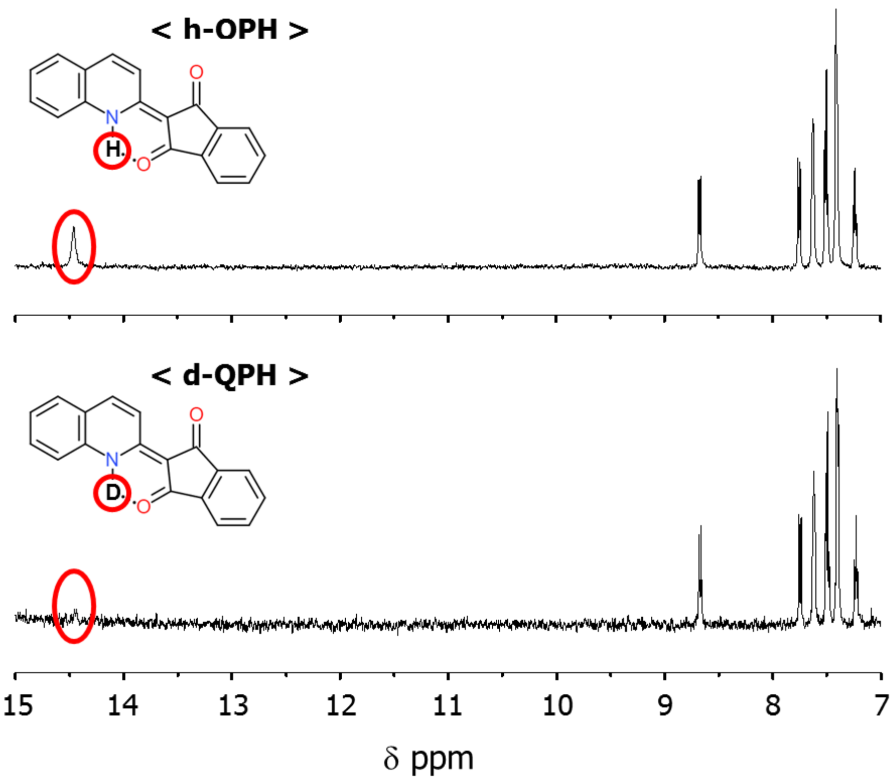

Figure S6. ^{1}H NMR spectrum of quinophthalone before and after deuterium substitution. ^{1}H NMR (500 MHz, d-C_6H_{12}, 25℃, anhydrous quinophthalone) δ=14.46 (s, 1H), δ=8.71-8.64 (d, 1H), δ=7.66-7.58 (d, 1H), δ=7.62 (s, 2H), δ=7.54-7.45 (m, 2H), δ=7.44-7.35 (m, 3H), δ=7.26-7.18 (m, 1H).

Table S1. Calculated energy levels and oscillator strength of relevant excited state geometries of quinophthalone.

Structure	Energy (Hartree)		Relative Energy (eV)		Oscillator strength	
P_E	S_0	-897.2539076186	S_0	0		
	S_1	-897.1385453346	S_1	3.139167	S_1	0.425
	S_2	-897.1330753568	S_2	3.288013	S_2	0.057
P_{E-S1}	S_0	-897.2418669059	S_0	0.327644		
	S_1	-897.1480129382	S_1	2.88154	S_1	0.263
	S_2	-897.1344058189	S_2	3.251809	S_2	0.000
$P_{E-S2/S1(CI)}$	S_0	-897.2419773396	S_0	0.324639		
	S_1	-897.1390621359	S_1	3.125104	S_1	0.009
	S_2	-897.1390617732	S_2	3.125114	S_2	0.000
P_{E-S2}	S_0	-897.2337258860	S_0	0.549173		
	S_1	-897.1537378300	S_1	2.725758	S_1	0.007
	S_2	-897.1319549148	S_2	3.318501	S_2	0.000
$P_{E/K(TS)}$	S_0	-897.2207032385	S_0	0.903537		
	S_1	-897.1489428020	S_1	2.856238	S_1	0.016
	S_2	-897.1196093979	S_2	3.65444	S_2	0.000
P_K	S_0	-897.2219337797	S_0	0.870052		
	T_1	-897.1745353123	T_1	2.15983		
	S_1	-897.1562850009	S_1	2.656446	S_1	0.032
	T_2	-897.1332420825	T_2	3.283476		
	S_2	-897.1223280631	S_2	3.580461	S_2	0.000

Table S2. Coordinates of optimized geometries of quinophthalone at B3LYP/6-31G(d) level.

We 'scanned' the S_1 potential surface by simulating the dynamics (B3LYP/TDDFT) for 2.5ps, starting from the Franck-Condon point. By analyzing the trajectory that shows ESIPT, we found that the molecule took some time vibrating in three major conformations. We performed optimization for different geometries at selected timestamps and found the geometry undergoing change in following order: **P_E**, **$P_{E\text{-}S1}$**, **$P_{E\text{-}S2}$** and **P_K**. Although the simulation played crucial role in identifying the geometrical points, we did not handle it in the main article because the calculation was performed without fully considering nonadiabatic coupling terms and transition probability. A dynamics study of QPH excited state is under way to address finer details.

1. **P_E** (Franck-Condon point)

C	-0.3763622187	0.0734213637	-0.0275331859
C	0.8975570987	-0.4714479089	-0.0590573306
C	-0.5648660003	1.4680442717	0.0134860274
C	0.5327948691	2.3122166623	0.0213845542
C	1.8430972364	1.7914065922	-0.0072261492
C	2.0154774897	0.3831156315	-0.0462197376
C	3.0244595982	2.6072123100	0.0072291597
C	4.2717525227	2.0594326693	-0.0032456821
C	4.4336729782	0.6313661154	-0.0324034156
N	3.2943284931	-0.1242980718	-0.0640691853
C	5.6693303737	-0.0282707100	-0.0231655158
C	5.8044965532	-1.4748586929	-0.0443441366
C	7.0002546475	0.5831444724	0.0379265880
C	7.9746107346	-0.5658404852	0.0591321666
C	7.2688823952	-1.7757390123	0.0057624347
C	9.3606712620	-0.5461093919	0.1245139124
C	10.0349364852	-1.7753022442	0.1329735168
C	9.3292088865	-2.9839783368	0.0760322670
C	7.9289596509	-2.9971715605	0.0116913475
O	7.3115795267	1.7720175231	0.0699936446
O	4.8928385227	-2.3190316112	-0.0901987817
H	-1.2389776981	-0.5872065122	-0.0329053750
H	1.0452738966	-1.5477957645	-0.0888354749
H	-1.5698104504	1.8789995885	0.0396511249
H	0.3993710611	3.3909086264	0.0526335833
H	2.9054897844	3.6880547355	0.0308534619
H	5.1764411491	2.6552261454	0.0142830862
H	3.4640862928	-1.1411228163	-0.0864429095
H	9.8959453622	0.3982139142	0.1692576702
H	11.1207001751	-1.7932910614	0.1853211169
H	9.8766644969	-3.9230692105	0.0830473211
H	7.3699848252	-3.9276972312	-0.0314361028

2. **$P_{E\text{-}S1}$**

C	-0.3387820947	0.0014582935	-0.0588984434
C	0.9663304250	-0.4832530047	-0.0467299354
C	-0.5658074345	1.4004138294	-0.0437961856
C	0.4766213560	2.3001192404	-0.0172209790
C	1.8403421557	1.8486712345	-0.0037703308
C	2.0395542277	0.4152189638	-0.0199161980
C	2.9582039101	2.6765031532	0.0233468018
C	4.2865084706	2.1359730202	0.0348873258
C	4.4343851283	0.7612986124	0.0189693244
N	3.3391035583	-0.0449381575	-0.0075911891

C	5.6953611556	0.0029628668	0.0289999150
C	5.8003383775	-1.4516792386	0.0051644208
C	7.0381272240	0.5810906647	0.0647317232
C	7.9913412775	-0.5802109436	0.0631840034
C	7.2555994980	-1.7763043485	0.0280524390
C	9.3777910824	-0.5896354014	0.0903419206
C	10.0226258449	-1.8352019999	0.0817754923
C	9.2875504020	-3.0298055359	0.0466047216
C	7.8868160734	-3.0137619998	0.0191568055
O	7.3527247293	1.7696425313	0.0897472121
O	4.8793931814	-2.2871046393	-0.0267117550
H	-1.1767297214	-0.6877152147	-0.0798671580
H	1.1665910409	-1.5523342613	-0.0579410330
H	-1.5884302459	1.7703610264	-0.0535027401
H	0.2852676606	3.3698387619	-0.0060275567
H	2.8210966139	3.7538768232	0.0364642321
H	5.1651852033	2.7628319680	0.0568596172
H	3.5305735845	-1.0598392562	-0.0209644857
H	9.9363425327	0.3413985871	0.1177506252
H	11.1084056312	-1.8785876822	0.1028839501
H	9.8148209139	-3.9800360150	0.0405684680
H	7.3055982372	-3.9307018785	-0.0084610082

3. **P**$_{\mathbf{E\text{-}S1/S2(CI)}}$

C	-0.3475138169	0.2996098195	-0.0472363937
C	0.9078370911	-0.2897202941	-0.0688545204
C	-0.4927746048	1.6831120158	0.1626976796
C	0.6298007586	2.4732814884	0.3496726966
C	1.9203120566	1.9057735933	0.3327046244
C	2.0495342783	0.5091666961	0.1219659495
C	3.1267899676	2.6609164920	0.5180246571
C	4.3521420703	2.0648874188	0.4959991427
C	4.4758541460	0.6490634062	0.2882204905
N	3.3095924973	-0.0463347815	0.1095822903
C	5.6829277789	-0.0651569832	0.2604157919
C	5.7831104661	-1.5381416614	0.0617779003
C	7.0318789814	0.4282017139	0.4243221656
C	7.9444061134	-0.6762316321	0.3362695718
C	7.1952713395	-1.8738267852	0.1168756197
C	9.3485719754	-0.7253418535	0.4342360831
C	9.9720306403	-1.9599738645	0.3144397036
C	9.2299315882	-3.1473566477	0.0978881504
C	7.8409663966	-3.1042404422	-0.0015222309
O	7.3005226141	1.6749114736	0.6168926110
O	4.7906171186	-2.2789082152	-0.1103650188
H	-1.2286131735	-0.3189995422	-0.1937614802
H	1.0207419741	-1.3584272596	-0.2306527998
H	-1.4831058404	2.1283546302	0.1769736395
H	0.5317636872	3.5436630603	0.5130014417
H	3.0488504224	3.7332833191	0.6795308448
H	5.2629534855	2.6359017061	0.6362653200
H	3.4388015313	-1.0580647785	-0.0326376532
H	9.9284022488	0.1779007534	0.6009155164
H	11.0550951025	-2.0171574280	0.3885863261
H	9.7509686718	-4.0963381239	0.0096311581
H	7.2569598290	-4.0059885178	-0.1674800165

4. **P**$_{\mathbf{E\text{-}S2}}$

C	-4.9358107884	1.6042329011	0.0001414505
C	-3.5585911682	1.6900645511	-0.0001387936
C	-5.5841920287	0.3474864630	0.0004547860
C	-4.8419808141	-0.8202905938	0.0004749347
C	-3.4322624219	-0.7778744728	0.0001919863
C	-2.7897957026	0.5021672080	-0.0001073124
C	-2.6043990971	-1.9475288525	0.0001944049
C	-1.2417096167	-1.8533235358	-0.0000507741
C	-0.6185117498	-0.5595653021	-0.0002936281
N	-1.4336930087	0.5527473902	-0.0003429965
C	0.7490059592	-0.3357626874	-0.0004062157
C	1.3551756964	1.0561968562	-0.0003826116
C	1.8583521052	-1.3716743003	-0.0002624480
C	3.0549601499	-0.5846208316	-0.0000300439
C	2.7556714321	0.8453712959	-0.0000963411
C	4.4039073705	-1.0122280021	0.0003002809
C	5.4070125891	-0.0702645699	0.0005686321
C	5.1099534922	1.3396262783	0.0005125948
C	3.8125321311	1.7908443277	0.0001795110
O	1.6953960590	-2.6063330259	-0.0002969721
O	0.6719404793	2.1181775360	-0.0005314504
H	-5.5296156894	2.5137184054	0.0001239885
H	-3.0519926306	2.6508439604	-0.0003726161
H	-6.6690869390	0.3030631215	0.0006775847
H	-5.3351073916	-1.7887208732	0.0007073779
H	-3.0852008136	-2.9225248711	0.0004060988
H	-0.5761165700	-2.7098170223	-0.0000424377
H	-0.8847662829	1.4511519785	-0.0004860758
H	4.6245404216	-2.0763628416	0.0003464980
H	6.4476642853	-0.3842493797	0.0008329400
H	5.9336242633	2.0487899997	0.0007378785
H	3.5805858953	2.8525087298	0.0001386279

5. **P**$_{\mathbf{E/K(TS)}}$

C	4.8644368214	1.6554030493	0.0014650679
C	3.4877994662	1.7075614535	0.0020001784
C	5.5432000291	0.4125030453	0.0016388125
C	4.8296514552	-0.7718006349	0.0023930352
C	3.4186118585	-0.7627702289	0.0030343742
C	2.7352264938	0.5023014725	0.0027871657
C	2.6270145063	-1.9541144416	0.0039450041
C	1.2629457616	-1.8894580872	0.0045773786
C	0.6288659170	-0.6016013425	0.0041414824
N	1.3845938866	0.5419363597	0.0033125607
C	-0.7505877384	-0.3993341405	0.0044541759
C	-1.3148863174	0.9782914368	0.0035496917
C	-1.8685816399	-1.3997410445	0.0052087327
C	-3.0505264235	-0.5804427467	0.0044769537
C	-2.7157551839	0.8444286909	0.0035461876
C	-4.4084165609	-0.9704921867	0.0046133911
C	-5.3851818035	0.0008084827	0.0038043288
C	-5.0543166100	1.4008106505	0.0028261484
C	-3.7453335587	1.8200279392	0.0026408957
O	-1.7463079176	-2.6414330829	0.0061209462

O	-0.5640227054	2.0271461197	0.0029428517
H	5.4381688788	2.5778996647	0.0008903687
H	2.9573619751	2.6550755782	0.0018540935
H	6.6291357642	0.3937245253	0.0011831436
H	5.3466787567	-1.7280926328	0.0025460095
H	3.1334461443	-2.9163907256	0.0041755809
H	0.6148744681	-2.7592349895	0.0053176277
H	0.5822917272	1.5055496687	0.0030647781
H	-4.6565716811	-2.0282501445	0.0053104605
H	-6.4340156038	-0.2853013981	0.0039138834
H	-5.8596360259	2.1303847950	0.0021739028
H	-3.4897951525	2.8760816884	0.0018625856

6. $\mathbf{P_K}$

C	-4.9467025093	1.5801341514	0.0001222972
C	-3.5749052863	1.6704616410	-0.0001499584
C	-5.5854394918	0.3137433469	0.0004372747
C	-4.8366271561	-0.8484324064	0.0004572214
C	-3.4263002581	-0.7956854004	0.0001770942
C	-2.7742326033	0.4890983510	-0.0001131572
C	-2.5956861283	-1.9548487532	0.0001761236
C	-1.2358896254	-1.8292714440	-0.0000628261
C	-0.6564229731	-0.5112266688	-0.0002791518
N	-1.4324723910	0.6079450630	-0.0003215915
C	0.7419620461	-0.3190883790	-0.0003676668
C	1.3699529602	0.9987546950	-0.0003382050
C	1.8237921157	-1.3560023395	-0.0002283032
C	3.0437214593	-0.5908219928	-0.0000241476
C	2.7609001426	0.8400922040	-0.0000908477
C	4.3859569527	-1.0237355431	0.0002779678
C	5.3919770249	-0.0792845615	0.0005172187
C	5.1095083208	1.3284927059	0.0004540165
C	3.8134310000	1.7888971490	0.0001445428
O	1.6761895288	-2.5996857047	-0.0002394750
O	0.7187046559	2.1502332014	-0.0004776391
H	-5.5505729933	2.4833293039	0.0001015057
H	-3.0683951090	2.6309753815	-0.0003821720
H	-6.6704812805	0.2609812223	0.0006596710
H	-5.3252937715	-1.8197347382	0.0006862790
H	-3.0618086447	-2.9376526061	0.0003749421
H	-0.5532872264	-2.6723031781	-0.0000644769
H	-0.2566968811	1.9007249636	-0.0006304358
H	4.6031785617	-2.0880000314	0.0003305455
H	6.4312791856	-0.3987219566	0.0007653616
H	5.9378637256	2.0313280805	0.0006540907
H	3.5898628158	2.8520490165	0.0000986313
